# Cases from My Note-book

**Published:** 1883-03

**Authors:** Julius Schmitt

**Affiliations:** Rochester, N. Y.


### CASES FROM MY NOTE-BOOK.

JULIUS SCHMITT, M. D., ROCHESTER, N. Y.

CASE I.—25th of May, 1882. William R., 14½ *years old*, blonde, fair complexion, of spare habit and *rather tall for his age*, whose mother died of typhoid fever, father unknown; has been sick for the last two months, and grew worse right along under "scientific" treatment. He complains of pains in right and left hypochondria and abdomen; *all these parts very sore to the least touch.* There is dropsical effusion in the peritoneum; thin, watery, yellow stools, but painless, three or four times a day, which hurry him to the closet and weaken him very much.

*Face pale and somewhat bloated; his whole skin has a milky-blue appearance;* pulse 120.

Lips dark red; tongue coated with a *fine, dried, red, varnish-like skin.* Appetite very poor, great thirst for *very cold drinks*, especially at night on account of dryness of mouth, tongue, and throat.

He cannot sleep at night on account of a sensation as if an aching ball were whirling around navel. (Patient's own words.)

He notices this sensation only at night when lying down, not so much in the daytime.

*Burning on outside of abdomen.*—Right-sided complete inguinal hernia; painful when coughing; dry cough; raises sometimes some white phlegm, more however by hawking, which he is impelled to do very often.

When he lies down in daytime the attendants notice a hectic flush and perspiration about head and neck, especially *when falling asleep*.

*Hands are bluish; feet œdematous; sore pain in soles of both feet when treading on them.*

Percussion showed enlargement of liver and spleen, which, in connection with the ascites and tenderness of abdomen and diarrhœa, pointing to some mesenteric gland trouble, made the prognosis rather unfavorable. Phosphorous$^{cm}$ (Swan); one dose dry on tongue and Placebos.

30th of May. To my astonishment he came to-day to my office, saying that he feels a great deal better.

Sensation of an aching ball whirling around navel entirely gone (was the most recent symptom).

Burning of skin of abdomen, better; bowels move once or twice in twenty-four hours, still the same in character; it drove him out of bed this morning (what a temptation to give Sulphur and spoil the case!); soles of feet not so sore; abdomen less tender; tongue more moist; pulse 114; thirst considerable yet; appetite not good.

A new symptom was the following: Pain in left lung when breathing deeply or lying on left side. Sacch. lactis.

7th of June. Feels better; appetite is increasing; very little tenderness of abdomen and both hypochondria; ascites hardly noticeable; sweats a good deal at night yet; bowels move once in twenty-four hours, watery, yellowish; the minute he gets out of bed he has to run; soles of feet not sore any more; soreness of left lung much less, but both lungs feel sore when breathing very hard; no cough; tongue moist, of more natural appearance; thirst considerably diminished; it is a great exertion for him to walk up hill or up stairs; his sleep is very bad, can hardly sleep at all; his strength is very poor yet; feels worse in rainy weather. Sacch. lact.

16th of June. Appetite very good now, but has to be careful not to eat too much; it will oppress the stomach, makes him feel bloated; better from walking around; sleep is better; sweat during every sleep; some slight pain in left chest when yawning, not otherwise any more.

Bowels the same as last time, but not the hurrying in the morning; pulse 96, feels stronger; tongue moist, with a yellow coating; some hungry sensation at 10½ A. M. and 4 P. M.

Milk acts on his bowels like a cathartic. Sacch. lact.

29th of June. Says he feels ever so much better. Appetite is very good; stomach tolerates food in large quantities. Sleep good; still perspiration during sleep. Pain in left chest when yawning. He feels stronger; tongue is clean and natural; no thirst any more. Bowels the same yet—face shows a little color. Soreness of abdomen almost gone, swelling entirely; swelling of feet has also disappeared. Sacch. lact.

11th of July. He feels still better. Appetite is very good; food, if taken in too large quantity, will distress him once in a while. Sweat during sleep; pain in chest is hardly noticed. Strength has improved considerably. Bowels continue to act more natural. Some slight soreness in both hypochondria yet, but none in abdomen. Sacch. lact.

14th of August. He had been gaining right along, and felt first-rate until the 11th of August, when a diarrhœa commenced, which lasted for two days. It was brought on by eating apple sauce, prepared from green apples. There was some blood in the stools; he had to hurry to the closet, but when at stool hardly anything would pass. Diarrhœa has stopped now, but his bowels and rectum feel sore, and the latter feels as if it was hanging down when he walks. He bloats after eating; taking something warm makes him belch and produces relief. Sulphur$^{30}$, one dose, and Sacch. lact.

10th of October. Has felt good right along. Bowels regular. Since a month a warm, strong-smelling foot-sweat appeared; he never had sweaty feet before. Otherwise no complaints. Sacch. lact.

I have seen the boy since several times; every time states that he feels good, even better than any time in his life before. Hernia remained as before, but has not been painful when coughing.

Case II.—Isaac C., a colored coachman, 39 years old, thin and of small stature, called at my office on the 15th of July, 1882. He states that he has been suffering from rheumatic pain for the last six months, and is unable to attend to his work.

Since four months he has a weak feeling in small of back; worse from cold air and when stooping.

If he stands any length of time a *numb sensation* creeps up from the knees to *penis and scrotum*, which ceases as soon as he sits down. (This symptom is the latest.)

For the last four weeks he notices a pain in left hypochondrium when lying down at night; after lying awhile it passes off; left hypochondrium sore to touch.

No appetite; if he feels like eating, has to do so right away or desire disappears.

Urine reddish and cloudy; micturition difficult when his back is very bad. Impotence, with no inclination for coitus. Warm night sweats.

Short, dry cough, not very troublesome, with sharp pains in both hypochondria. During the war he had a *suppuration of right submaxillary gland*, which had to be opened. Baryta. carb.$^{1000}$ one dose in the office, another one to-night.

Went off quite astonished not to receive more medicine. The remedy was selected from Allen's Symptom Register.

22d of July. Comes to office without his cane; regards himself better, but says that for the first three days, after taking the powders, he felt bad and weak all over.

Numb feeling of privates entirely gone; weakness of back better; micturition easier and urine clearer. Pain in left hypochondrium is less; appetite and night sweats very little better. One powder Sacch. lactis.

29th of July. Feels still better, stronger, appetite pretty good; had the numb feeling about the privates once last week, but it passed off very soon. Sacch. lact. one powder.

5th of August. Better; stronger about genitals; noticed return of sexual desire; appetite not quite good. Sacch. lact. one powder.

21st of August. Improvement of all his symptoms, but complains of a pain in left hip; he states, however, that this pain has troubled him right along, but he forgot to mention it. Sacch. lact. one powder.

11th of September. Left hip pained him much for the last week, and there are now also pains in flexor-tendons of left knee-joint. There is some stiffness in back yet, especially when rising from sitting. Appetite is not as good as it was and urine looks darker again. (There was an evident standstill in the improvement, but no change in the symptoms, which would indicate another remedy. The same remedy had to be given, but, according to Hahnemann, in a different potency.) Baryta. carb.$^{cm}$ one powder.

27th of September. Left hip is better, but since two days a diarrhœa, which commenced between 8 and 9 A. M., with very little crampy pains in abdomen; four very watery, yellow stools yesterday. Thirsty, but no appetite. His bowels have always been inclined to become loose. (If an old symptom appears, while the proper remedy is doing its work, Hahnemann teaches to leave it alone.) Sacch. lact. one powder.

19th of October. Diarrhœa soon got better, and he has not been troubled with it since. Appetite is very good; feels good all over. Once in a while he notices a little weakness in back yet. Pain in left hip almost gone; still some pain in left knee. Sacch. lact. one powder.

